# Personalized pulmonary vein isolation guided by left atrial wall thickness for persistent atrial fibrillation ablation: the PeAF-by-LAWT randomized trial

**DOI:** 10.1093/europace/euaf163

**Published:** 2025-08-06

**Authors:** Giulio Falasconi, Diego Penela, David Soto-Iglesias, Alessia Chiara Latini, Federico Landra, Emanuele Curti, Pietro Francia, Andrea Saglietto, Dario Turturiello, Daniel Viveros, Aldo Bellido, Jose Alderete, Fatima Zaraket, Paula Franco-Ocaña, Stefano Valcher, Francesco Amata, Chiara Valeriano, Carlo Gigante, Lucio Teresi, Bruno Tonello, Roberta Mea, Lautaro Sánchez-Mollá, Carmine De Lucia, Marina Huguet, Óscar Cámara, José-Tomás Ortiz-Pérez, Julio Martí-Almor, Antonio Berruezo

**Affiliations:** Arrhythmia Department, Teknon Medical Center, c. Villana, 12, Barcelona 08022, Spain; Facultat de Medicina i Ciències de la Salut, Universitat de Barcelona (UB), c. Casanova, 143, Barcelona 08036, Spain; Arrhythmology Department, Humanitas Research Hospital IRCCS, Rozzano, Milan, Italy; Department of Biomedical Sciences, Humanitas University, Pieve Emanuele, Milan, Italy; Arrhythmia Department, Teknon Medical Center, c. Villana, 12, Barcelona 08022, Spain; Arrhythmology Department, Humanitas Research Hospital IRCCS, Rozzano, Milan, Italy; Department of Biomedical Sciences, Humanitas University, Pieve Emanuele, Milan, Italy; Arrhythmia Department, Teknon Medical Center, c. Villana, 12, Barcelona 08022, Spain; Division of Cardiology, Università degli Studi di Siena, Viale Bracci 4, Siena 53100, Italy; Arrhythmia Department, Teknon Medical Center, c. Villana, 12, Barcelona 08022, Spain; Department of Medicine and Surgery, University of Milano-Bicocca, Milan, Italy; Arrhythmia Department, Teknon Medical Center, c. Villana, 12, Barcelona 08022, Spain; Department of Clinical and Molecular Medicine, St. Andrea Hospital, Sapienza University, Rome, Italy; Arrhythmia Department, Teknon Medical Center, c. Villana, 12, Barcelona 08022, Spain; Department of Medical Sciences, University of Turin, Turin, Italy; Arrhythmia Department, Teknon Medical Center, c. Villana, 12, Barcelona 08022, Spain; Arrhythmia Department, Hospital El Pilar, Barcelona, Spain; Arrhythmia Department, Teknon Medical Center, c. Villana, 12, Barcelona 08022, Spain; Facultat de Medicina i Ciències de la Salut, Universitat de Barcelona (UB), c. Casanova, 143, Barcelona 08036, Spain; Arrhythmia Department, Teknon Medical Center, c. Villana, 12, Barcelona 08022, Spain; Arrhythmia Department, Hospital El Pilar, Barcelona, Spain; Arrhythmia Department, Teknon Medical Center, c. Villana, 12, Barcelona 08022, Spain; Facultat de Medicina i Ciències de la Salut, Universitat de Barcelona (UB), c. Casanova, 143, Barcelona 08036, Spain; Arrhythmia Department, Teknon Medical Center, c. Villana, 12, Barcelona 08022, Spain; Arrhythmia Department, Teknon Medical Center, c. Villana, 12, Barcelona 08022, Spain; Arrhythmology Department, Humanitas Research Hospital IRCCS, Rozzano, Milan, Italy; Arrhythmology Department, Humanitas Research Hospital IRCCS, Rozzano, Milan, Italy; Department of Biomedical Sciences, Humanitas University, Pieve Emanuele, Milan, Italy; Arrhythmology Department, Humanitas Research Hospital IRCCS, Rozzano, Milan, Italy; Arrhythmia Department, Teknon Medical Center, c. Villana, 12, Barcelona 08022, Spain; Arrhythmia Department, Teknon Medical Center, c. Villana, 12, Barcelona 08022, Spain; Department of Clinical and Experimental Medicine, University of Messina, Messina, Italy; Arrhythmia Department, Teknon Medical Center, c. Villana, 12, Barcelona 08022, Spain; Arrhythmia Department, Teknon Medical Center, c. Villana, 12, Barcelona 08022, Spain; Department of Biomedical and Clinical Sciences, University of Milan, Milan, Italy; Arrhythmia Department, Teknon Medical Center, c. Villana, 12, Barcelona 08022, Spain; Arrhythmia Department, Teknon Medical Center, c. Villana, 12, Barcelona 08022, Spain; Arrhythmia Department, Teknon Medical Center, c. Villana, 12, Barcelona 08022, Spain; Department of Information and Communication Technologies, Pompeu Fabra University, Barcelona, Spain; Arrhythmia Department, Teknon Medical Center, c. Villana, 12, Barcelona 08022, Spain; Facultat de Medicina i Ciències de la Salut, Universitat de Barcelona (UB), c. Casanova, 143, Barcelona 08036, Spain; Arrhythmia Department, Teknon Medical Center, c. Villana, 12, Barcelona 08022, Spain; Arrhythmia Department, Teknon Medical Center, c. Villana, 12, Barcelona 08022, Spain

**Keywords:** Persistent atrial fibrillation, Left atrial wall thickness, Catheter ablation, Pulmonary vein isolation, Multidetector computed tomography

## Abstract

**Aims:**

A personalized pulmonary vein isolation (PVI) approach aimed at ablation index (AI) titration according to multidetector computed tomography-derived left atrial wall thickness (LAWT) maps reported high effectiveness and efficiency outcomes for persistent atrial fibrillation (PeAF) ablation. To date, no randomized trials have compared this approach with the standard CLOSE protocol. This non-inferiority randomized controlled trial sought to compare a LAWT-guided PVI with CLOSE protocol-based for PeAF (NCT05396534).

**Methods and results:**

Consecutive patients referred for first-time PeAF ablation were randomized on a 1:1 basis. In the by-LAWT arm, the AI was titrated according to local LAWT, and the ablation line was personalized to avoid the thickest regions at the pulmonary vein antrum. In the CLOSE arm, LAWT information was not available to the operator; the ablation was performed according to the CLOSE study settings: AI is ≥400 at the posterior wall and ≥550 at the anterior wall. Primary endpoint was freedom from atrial arrhythmias recurrence. Secondary endpoints were the major complication rate, procedure time, radiofrequency time, and first-pass PVI rate. One hundred fifty-six patients were included. At 12 month follow-up, no significant difference occurred in atrial arrhythmia-free survival between groups (*P* = 0.50). In the by-LAWT group, a significant reduction in procedure time (60.5 vs. 80.0 min; *P* < 0.01) and RF time (14.4 vs. 28.6 min; *P* < 0.01) was observed. No difference was observed regarding first-pass PVI (*P* = 0.72) and the major complication rate (*P* = 0.99).

**Conclusions:**

The PeAF-by-LAWT trial is the first prospective randomized study to demonstrate that a personalized LAWT-guided PVI for PeAF ablation is non-inferior to the standard CLOSE protocol in terms of arrhythmia-free survival while significantly improving procedural efficiency. The study was not powered to detect differences in safety outcomes.

## Graphical Abstract

**Graphical Abstract:**
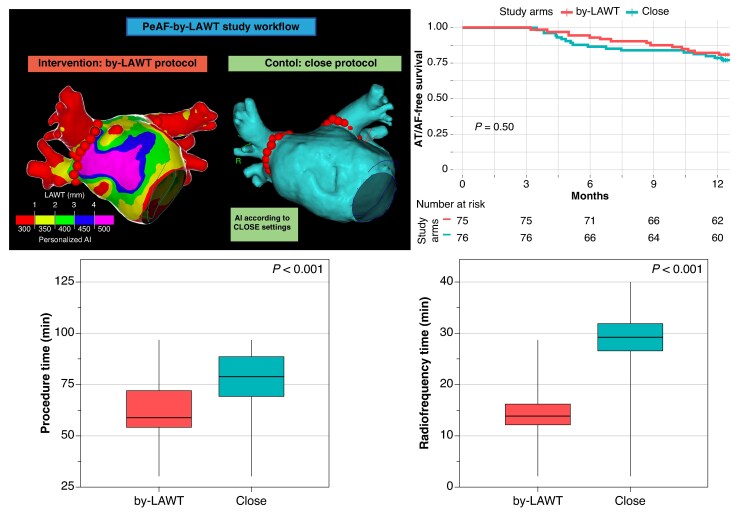

What’s new?

- The persistent atrial fibrillation (PeAF)-by-left atrial wall thickness (LAWT) is the first prospective randomized trial comparing the outcomes of a LAWT-guided pulmonary vein isolation (PVI) with the standard CLOSE protocol in a cohort of patients with PeAF.
- The LAWT-guided approach enables tailored radiofrequency titration based on local atrial wall thickness, allowing the operator to avoid the thickest left atrial regions and to reduce energy delivery in areas of close proximity between the posterior left atrial wall and the multidetector computed tomography-derived position of the oesophagus.
- The PeAF-by-LAWT randomized trial demonstrates that, in patients undergoing PVI for PeAF, the LAWT-guided strategy achieves non-inferior arrhythmia-free survival at 12 months compared to the CLOSE protocol while significantly reducing procedural and radiofrequency times.

## Introduction

Atrial fibrillation (AF) is the most common arrhythmia in the adult population^1,2^ and is associated with significant morbidity and mortality.^3^ Pulmonary vein isolation (PVI) has become the first-line approach of the ablation procedure and the cornerstone of the rhythm control strategy.^4^

AF is a progressive disease, with more than one-third of patients with paroxysmal AF progressing to persistent forms within 10 years.^5^ In patients with persistent AF (PeAF), PVI outcomes are significantly worse compared to those with paroxysmal AF.^6^ While additional ablation techniques—such as linear ablations, posterior wall box lesions, complex fractionated atrial electrogram ablation, and others—have been explored, they have not consistently improved long-term arrhythmia-free survival rates.^7,8^ Consequently, to date, PVI-only approach remains the mainstay therapy for PeAF.

Pulmonary vein (PV) reconnection is a common cause of AF recurrence, primarily attributed to non-contiguous or non-transmural radiofrequency lesions,^9^ particularly in thicker regions of the left atrium (LA). However, excessive radiofrequency delivery can increase complication rates, especially in thinner areas of the LA.^10^ To address this, the traditional approach involves dichotomizing the LA lesion set into posterior and anterior regions, with the aim of achieving greater radiofrequency application in the anterior region and less in the posterior. Building on this concept, the CLOSE study proposed a refined ablation strategy targeting an interlesion distance (ILD) of ≤6 mm and ablation index (AI) values of 400 for the posterior/inferior segments of the PV antrum and 550 for the anterior/superior segments.^11^

However, imaging and histological studies reveal that the LA is a complex structure, with wall thickness ranging from 0.5 to 5 mm, exhibiting significant inter- and intrapatient variability. It has been recently shown that pre-procedural multidetector computed tomography (MDCT)-derived data can be post-processed to obtain 3D left atrial wall thickness (LAWT) maps.^12^ Integrating these LAWT maps into the navigation system provides a real-time knowledge of the local LAWT in contact with the ablation catheter tip during the ablation, permitting a personalized LAWT-guided PVI approach aimed to an AI titration according to the local LAWT. The proposed approach demonstrated to be safe and to achieve a high arrhythmia-free survival rate at 12 months following the ablation procedure in PAF and PeAF patients.^13–15^ However, there are no randomized trials comparing the outcomes of a LAWT-guided PVI approach with those of the standard CLOSE protocol in PeAF patients.

The present randomized controlled trial was designed as a non-inferiority study to compare the 12 month procedural effectiveness of a LAWT-guided personalized PVI approach vs. a standard CLOSE protocol in patients with PeAF while also evaluating differences in procedural efficiency between the two strategies.

## Methods

### Patient sample

We conducted a two-arm, single-blind, multicentre, non-inferiority, prospective, randomized controlled trial (NCT05396534). Consecutive patients who underwent first-time PeAF ablation were prospectively enrolled between July 2022 and September 2023 in three Spanish hospitals with electrophysiology operators with proven experience in performing AF ablation. All patients had documented symptomatic PeAF and indication for ablation in accordance with ESC guidelines.^16^ As part of the routine clinical practice of the centres,^17^ an MDCT study was obtained in all patients prior to the ablation procedure. Exclusion criteria included age below 18 years, a history of prior AF ablation, inability to perform a pre-procedural MDCT, any clinical condition precluding general anaesthesia or high-frequency low-tidal volume (HFLTV) ventilation, and failure to provide signed informed consent. Patients meeting the inclusion criteria were consecutively enrolled and randomly assigned in a 1:1 ratio to either the study arm (‘by-LAWT’ arm) or the control arm (‘CLOSE’ arm). For allocation of the participants, a computer-generated list of random numbers was used; the randomization sequence was created using Excel 2020 (Microsoft, Redmond, WA, USA) with a 1:1 allocation. Written informed consent was obtained from all patients. The study complied with the Declaration of Helsinki and was approved by the local Institutional Ethics Committees.

**Figure 1:**
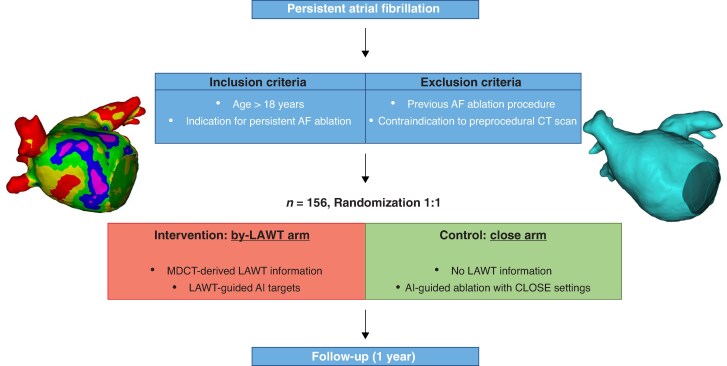
PeAF-by-LAWT randomized trial flowchart.

The study flowchart of the PeAF-by-LAWT study was reported in *Figure 1*.

### Pre-procedural image acquisition and post-processing

MDCT scans were acquired during an inspiratory breath-hold with a retrospective electrocardiogram (ECG)-gating technique. In the ‘by-LAWT’ arm, the MDCT-derived images were post-processed by two experienced biomedical engineers using the ADAS 3D^TM^ software (Adas3D Medical SL, Barcelona, Spain) to generate 3D LAWT maps.^12,18–20^ LAWT was calculated using a semi-automated threshold-based method supported by artificial intelligence: the endocardial surface was semi-automatically delineated, while the epicardial surface was automatically defined through an artificial intelligence-based segmentation pipeline integrated into the software, with the option for manual adjustments by the operator. Finally, LAWT was automatically calculated at each point as the distance between the endocardial surface and its corresponding projection on the epicardial surface. The resulting LAWT maps were colour-coded as follows: red for LAWT < 1 mm, yellow for LAWT between 1 and 2 mm, green for LAWT between 2 and 3 mm, blue for LAWT between 3 and 4 mm, and purple for LAWT ≥ 4 mm. The mean time required for the image post-processing step was 10 ± 2 min per patient. Data on the reproducibility of the segmentation method have been reported previously^21^. Finally, a MATLAB-customized software was used to divide the circumference of both left pulmonary vein (LPV) and right pulmonary vein (RPV) antra in an eight-segment model, as previously described.^12,14^

### Ablation protocol based on the assigned study arm

All procedures were performed using CARTO3 mapping system (Biosense Webster, Johnson & Johnson Medical S.p.A., CA, USA) and were carried out under general anaesthesia and adopting an HFLTV ventilation protocol.^22^ The transseptal puncture was guided by transoesophageal echocardiography.^23^ All procedures were performed with single catheter technique,^24^ utilizing a contact force-sensing irrigated ablation catheter (Thermocool Smarttouch, Biosense Webster Inc., CA, USA). A fast anatomical map (FAM) of the entire LA anatomy and the PVs was acquired and then merged with the imported MDCT-derived information within the spatial reference coordinates of the CARTO system. Before starting ablation, a multi-thermocouple temperature probe (SensiTherm, St. Jude Medical, Inc., St. Paul, MN, USA) was always advanced into the oesophagus under fluoroscopic guidance to monitor the oesophageal temperature rises.

In the ‘by-LAWT arm’, PVI was performed following a point-by-point wide antral circumferential ablation (WACA) pattern, with a maximal ILD of 6 mm. The PVI lines were personalized, being moved inwards or outwards, aiming to avoid areas with thicker LAWT to perform ablation. The ablation lines were also tailored according to the imported oesophageal isodistance colour-coded map to avoid, as far as possible, radiofrequency application on the LA posterior wall where the MDCT-derived atrio-oesophageal distance is the shortest (<1 mm, the red area of the colour-coded map). The power output was set at 35 W for the posterior wall, 40 W for the anterior wall at zones with LAWT ≤2 mm (red and yellow zones on the thickness map), and 50 W anterior wall at zones with LAWT >2 mm (green, blue, and purple zones on the thickness map). AI targets were titrated according to the local thickness of the 3D LAWT map, as previously described^12–15^; briefly, the following AI targets were used: 300 for LAWT <1 mm (red zones), 350 for LAWT ≥1 mm and <2 mm (yellow zones), 400 for LAWT ≥2 mm and <3 mm (green zones), 450 for LAWT ≥3 mm and <4 mm (blue zones), and 500 for LAWT ≥4 mm (purple zones). A carina line was mandatory for the RPVs, except if a common trunk was found, as the RPV intervenous carina is a common site for both acute and late reconnections.^25^ Regardless of the local thickness of the 3D LAWT map, AI was down-titrated to 300 whenever the radiofrequency point was at <1 mm distance from the oesophagus footprint on the posterior LA wall (‘red zones’ of the oesophagus–LA isodistance map).^26^

In the ‘CLOSE arm’, the MDCT-derived anatomy was available for the operator without the LAWT information. PVI was performed following a point-by-point WACA pattern with a maximal ILD of 6 mm and using the CLOSE protocol settings^11,27^: maximum power of 35 W, temperature limit of 45°C, and CF > 5 g. Radiofrequency delivery aimed for an AI target of ≥400 at the posterior/inferior wall and ≥550 at the anterior/superior wall.

The ablation parameters of the study arms are summarized in *Table 1*.

**Table 1:** Ablation parameters of the study arms

By-LAWT arm					CLOSE arm		
LAWT	Colour code	Ablation Index	RF energy (watts)		Ablation Index		RF energy (watts)
			Anterior	Posterior	Anterior	Posterior	
< 1 mm	Red	300	40	35	550	400	35
1–2 mm	Yellow	350	40	35	550	400	35
2–3 mm	Green	400	50	35	550	400	35
3–4 mm	Blue	450	50	35	550	400	35
> 4 mm	Purple	500	50	35	550	400	35

LAWT, left atrial wall thickness; RF, radiofrequency.

In all cases, PVI was the only lesion set performed in all patients included in the study, regardless of the assigned ablation strategy. No additional ablation lesions beyond WACA were delivered. For both arms, PVI was performed during the presenting rhythm at the time of the procedure (either sinus rhythm or AF). As per the routine clinical practice at the centres, no AF induction was attempted at any time, neither via aggressive atrial stimulation nor by isoproterenol infusion. In patients who were in AF at the end of the WACA, electrical cardioversion was performed to confirm bidirectional PV isolation or to target residual PV–LA conduction gap during sinus rhythm. For both the ablation arms, acute PVI was confirmed by demonstrating bidirectional block: entry block was demonstrated by the absence of PV potentials inside the vein with the ablation catheter placed sequentially in each segment inside the circumferential PV line and exit block by proving the absence of electric capture of the atrium during high-output pacing (10 mA at 2 ms) from inside the circumferential PV line at multiple locations.^24^ Additional ‘touch-up’ applications were delivered at the earliest local EGM in the case of non-first-pass isolation or acute PV reconnection until PVI was achieved. The procedure was not terminated until confirming the absence of visual gaps between VisiTags.

**Figure 2:**
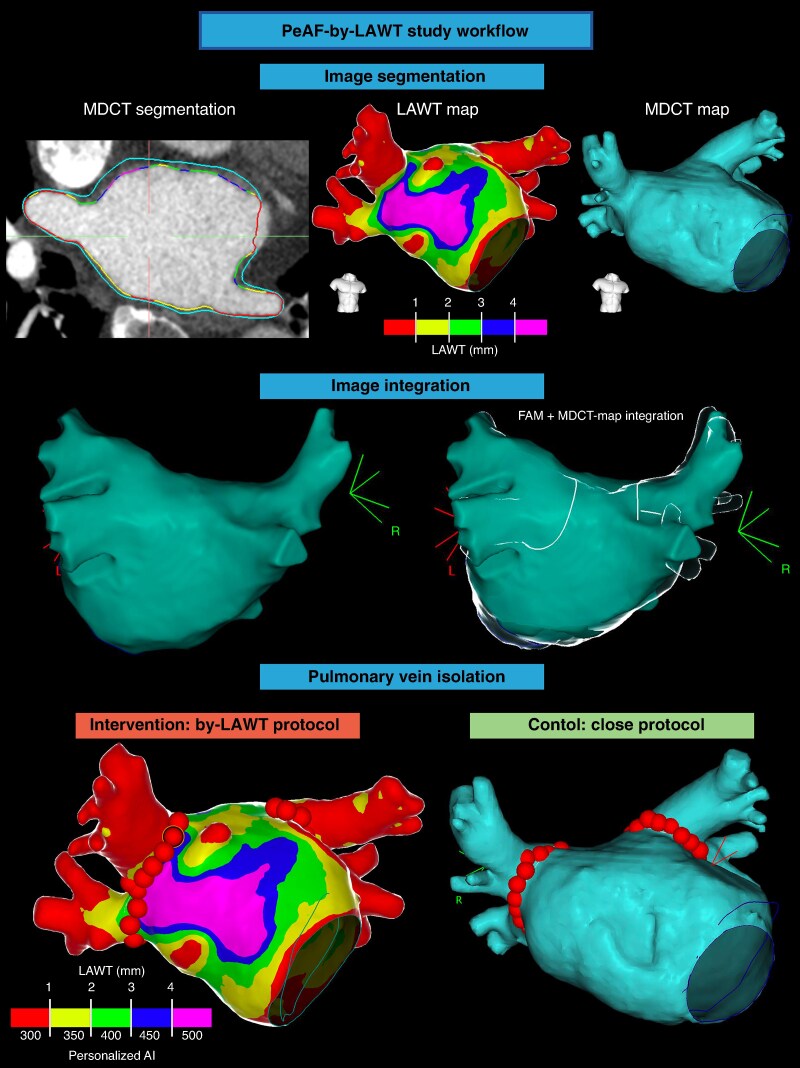
PeAF-by-LAWT study workflow. (*A*) The first step is the multidetector computed tomography (MDCT)-derived image segmentation and the rendering of the 3D colour-coded LAWT map (by-LAWT arm) or 3D LA anatomical map (CLOSE arm). (*B*) Image integration into the navigation system after performing LA fast electro-anatomical map. (*C*) Pulmonary vein isolation with AI targets adapted to LAWT information (by-LAWT arm) or with according to the CLOSE protocol (CLOSE arm).

**Figure 3:**
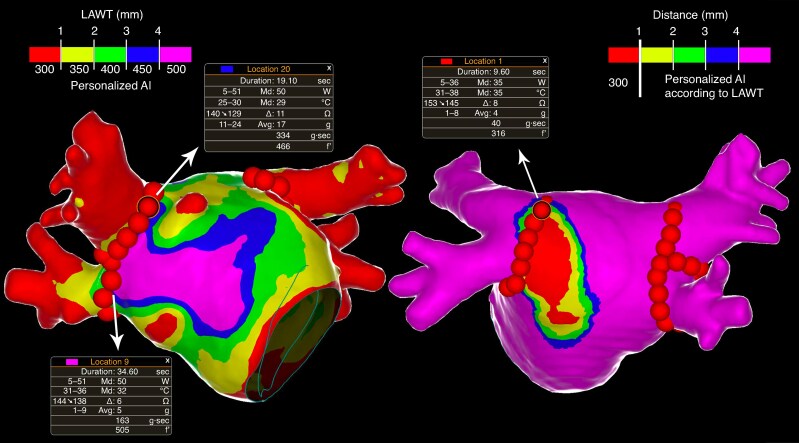
Personalized pulmonary vein isolation with the local LAWT-guided AI target. At the left, an example of a LAWT-guided PVI. The LAWT map is colour-coded in the following manner, as described in the main text: red for LAWT < 1 mm, yellow for LAWT between 1 and 2 mm, green for LAWT between 2 and 3 mm, blue for LAWT between 3 and 4 mm, and purple for LAWT ≥ 4 mm. AI targets were adjusted according to the underlying LAWT in a point-by-point fashion. At the right, an example of a personalized PVI line aiming at avoiding, as far as possible, the closest area (red area) of the oesophagus fingerprint at LA posterior wall; in addition, the AI target in correspondence to the red area of the oesophagus fingerprint was limited to 300.

The study workflow and the personalized LAWT-guided PVI approach are represented in *Figures 2* and *3.*

### Follow-up and study endpoints

Patients underwent a minimum clinical follow-up of 1 year, with scheduled visits at 1, 3, 6, and 12 months during the first year and annually thereafter in the absence of other clinical indications or symptoms. Follow-up visits were anticipated if symptoms occurred or if deemed necessary based on clinical judgement. Each evaluation included an ECG and 24 h Holter ECG monitoring. The primary outcome measure was freedom from atrial arrhythmias recurrence after a blanking period of 3 months. Recurrence was defined as any documented episode of AF, atrial flutter, or atrial tachycardia (AT) lasting more than 30 s, regardless of symptoms.^28^ All antiarrhythmic drugs (AADs) were discontinued at the end of the blanking period unless otherwise indicated; however, the decision to withdraw AADs was ultimately left to the discretion of the treating physician, clear recommendation to discontinue AADs after the 3 month blanking period in all patients, except for those who experienced arrhythmic recurrence during that period, in whom continuation of AAD was permitted. The secondary endpoints were procedure time, radiofrequency time, fluoroscopy time, and the first-pass PVI rate. The safety endpoint was freedom from serious adverse events, defined as a procedural-related event resulting in death or permanent injury, requiring an intervention for treatment or requiring prolonged hospitalization for more than 24 h.

### Sample size and statistical analysis

The sample size calculation was based on the assumption that the proposed LAWT-guided PVI approach (by-LAWT arm) would be an acceptable alternative to the CLOSE protocol, following a non-inferiority design. A non-inferiority margin of 10% in clinical success (defined as arrhythmia-free survival at 1 year follow-up post-ablation) was deemed the maximum acceptable difference between the two arms. A 5% dropout rate was anticipated and incorporated into the sample size estimation. A total of *n* = 156 participants (78 per arm) was calculated to provide 90% power to reject the null hypothesis of inferiority.

Continuous variables were presented as mean ± standard deviation or median (interquartile range) as appropriate. Categorical variables were reported as the total number (percentage). Student’s *t*-test or the Wilcoxon test was used to compare continuous variables, as appropriate; *χ*^2^ or Fisher’s exact test was used to compare categorical variables, as appropriate. Kaplan–Meier curves and the log-rank test were used to assess the cumulative AF-free survival. A level of *P* < 0.05 was considered statistically significant. Data were analysed with the R version 3.6.2 software (R Foundation for Statistical Computing, Vienna, Austria) and MATLAB statistics toolbox (MATLAB R2010a, The MathWorks, Inc., Natick, MA, USA).

## Results

### Baseline population

Between July 2022 and September 2023, a total of 156 consecutive patients were enrolled and randomized. Baseline clinical characteristics were balanced between groups (*Table 2*). In the whole cohort, mean age was 65.6 ± 10.3 years, and 69.2% were male. The 35.9% of the population had a prior diagnosis of structural heart disease, being hypertensive cardiomyopathy the most prevalent (16.0%); mean indexed LA volume was 45.2 ± 16.3 mL/m^2^, without significant differences between the two groups (*P* = 0.55).

**Table 2:** Patients’ baseline characteristics according to the study arm

	By-LAWT (*N* = 78)	CLOSE (*N* = 78)	Total patients (*N* = 156)	*P* value
Age (years)	65.4 ± 11.6	65.9 ± 9.0	65.6 ± 10.3	0.91
Male	53 (67.9)	55 (70.5)	108 (69.2)	0.84
BMI (kg/m^2^)	28.1 ± 5.5	29.6 ± 4.7	28.8 ± 5.2	0.13
Hypertension	43 (55.1)	46 (58.9)	89 (57.0)	0.71
Dyslipidaemia	20 (25.6)	13 (16.7)	33 (21.1)	0.25
Smoke history	4 (5.1)	10 (12.8)	14 (9.0)	0.20
Type 2 diabetes	8 (10.3)	8 (10.3)	16 (10.3)	0.99
LVEF	54.7 ± 7.4	53.7 ± 5.6	54.2 ± 6.6	0.44
LA diameter (mm)	43.3 ± 5.9	44.7 ± 5.7	44.0 ± 5.8	0.14
Indexed LA volume (mL/m²)	44.9 ± 14.9	45.4 ± 18.0	45.2 ± 16.3	0.55
CHA_2_DS_2_-VASc score	2.1 ± 1.5	2.2 ± 1.5	2.2 ± 1.5	0.70
Time between diagnosis to ablation				0.91
< 3 months	28 (35.9)	26 (33.3)	54 (34.6)	
3–12 months	28 (35.9)	32 (41.0)	60 (38.5)	
>12 months	22 (28.2)	20 (25.6)	42 (26.9)	
Underlying cardiomyopathy				0.91
None	51 (65.4)	49 (62.8)	100 (64.1)	
Hypertensive	13 (16.7)	12 (15.4)	25 (16.0)	
Ischaemic	6 (7.7)	4 (5.1)	10 (6.4)	
Non-ischaemic dilated	2 (2.6)	2 (2.6)	4 (2.6)	
Hypertrophic	0 (0.0)	1 (1.3)	1 (0.6)	
Valvular	3 (3.8)	4 (5.1)	7 (4.5)	
Other	3 (3.8)	6 (7.7)	9 (5.8)	

Results are reported as *n* (%) for categorical variables and median (interquartile range) or mean ± standard deviation for continuous variables.

BMI, body mass index; LA, left atrium; LVEF, left ventricle ejection fraction; PVs, pulmonary veins.

Patients’ baseline characteristics are summarized in *Table 1.*

### Procedural characteristics

Procedural results are summarized in *Table 3.*

**Table 3:** Procedural data according to the study arm

	By-LAWT (*N* = 78)	CLOSE (*N* = 78)	Total patients (*N* = 156)	*P* value
AF at the beginning of the procedure	50 (64.1)	43 (55.1)	93 (59.6)	0.33
Ventilation rate (breaths/min)	50 (45–50)	50 (45–50)	50 (45–50)	0.70
Tidal volume (ml)	250 (210–300)	250 (240–300)	250 (220–300)	0.61
Procedure time skin-to-skin (min)	60.5 (55.0–73.0)	80.0 (70.0–90.0)	70.0 (60.0–84.0)	**<0**.**01**
Fluoroscopy time (s)	82.0 (45.0- 147.0)	86.0 (59.8–132.0)	84.0 (50.0–139.5)	0.40
Fluoroscopy dose (mGy)	6.6 (3.6–13.3)	7.7 (4.3–13.6)	7.2 (3.8–13.6)	0.50
Dose area product (Gy*cm2)	1.9 (0.9–3.5)	2.0 (1.1–4.1)	2.0 (1.0–3.6)	0.55
Total RF time (min)	14.4 (13.0–16.5)	28.6 (26.3–31.2)	19.5 (14.2–28.7)	**<0**.**01**
Right pulmonary veins				
RF time (min)	7.4 (6.6–8.5)	14.7 (12.1–16.7)	10.2 (7.2–14.7)	**<0**.**01**
First-pass isolation	64 (82.1)	60 (76.9)	124 (79.5)	0.55
Anterior right PVs antrum WT (mm)	1.61 (1.28–1.97)	1.60 (1.24–1.95)	1.60 (1.26–1.96)	0.39
AI delivery at anterior right PVs antrum	390 (374–411)	553 (535–565)	465 (434–481)	**<0**.**01**
Posterior right PVs antrum WT (mm)	1.11 (0.92–1.33)	1.11 (0.89–1.47)	1.11 (0.90–1.39)	0.48
AI delivery at posterior right PVs antrum	335 (320–352)	395 (375–425)	370 (344–390)	**<0**.**01**
Left pulmonary veins				
RF time (min)	7.0 (6.1–8.0)	13.8 (12.2–15.7)	10.1 (6.9–13.9)	**<0**.**01**
First-pass isolation	65 (83.3)	67 (85.9)	132 (84.6)	0.82
Anterior left PVs antrum WT (mm)	2.15 (1.57–2.74)	2.20 (1.66–2.84)	2.19 (1.57–2.83)	0.13
AI delivery at anterior left PVs antrum	422 (383–464)	555 (525–570)	488 (450–515)	**<0**.**01**
Posterior left PVs antrum WT (mm)	1.18 (1.06–1.31)	1.15 (1.02–1.33)	1.16 (1.04–1.32)	0.45
AI delivery at posterior left PVs antrum	331 (315–371)	380 (360–410)	360 (338–380)	**<0**.**01**
Acute procedural complication	1 (1.3)	2 (2.6)	3 (1.9)	0.99

Results are reported as *n* (%) for categorical variables and median (interquartile range) or mean ± standard deviation for continuous variables.

AI, ablation index; FAM, fast anatomical map; RF, radiofrequency; SR, sinus rhythm; WT, wall thickness.

At the time of the procedure, 93 patients (59.6%) were in AF, with no significant difference between the by-LAWT and CLOSE groups (*P* = 0.33). Total procedural skin-to-skin time was lower in the by-LAWT group compared to that in the CLOSE group [60.5 min (55.0–73.0) vs. 80.0 min (70.0–90.0), *P* < 0.001]. No significant differences were observed between the groups in total fluoroscopy time [82 s (45–147) vs. 86 s (60–132), *P* = 0.40], total emitted fluoroscopy dose (6.6 mGy (3.6–13.3) vs. 7.7 mGy [4.3–13.6], *P* = 0.50), and dose area product [1.9 Gy*cm^2^ (0.9–3.5) vs. 2.0 Gy*cm^2^ (1.1–4.1), *P* = 0.55]. Median radiofrequency time was significantly lower in by-LAWT group with respect to the CLOSE group for the isolation of both LPVs [7.0 min (6.1–8.0) vs. 13.8 min (12.2–15.7), *P* < 0.001] and RPVs [7.4 min (6.6–8.5) vs. 14.7 min (12.1–16.7), *P* < 0.001]. No significant differences were observed in the first-pass isolation rate between the two groups for both LPVs (83.3 vs. 85.9%, *P* = 0.82) and RPVs (82.1 vs. 76.9%, *P* = 0.55). More in detail, 43 patients required additional ablation to achieve complete PVI (the absence of either RPVs or LPVs first-pass isolation): 20 patients (25.6%) in the by-LAWT group and 23 patients (29.5%) in the CLOSE group, with no significant difference between the groups (*P* = 0.72). Across these patients, a total of 66 PV–LA conduction gaps were identified, without statistically significant differences in the distribution of gap locations across the ablation line segments between the two groups (*P* = 0.76). In both groups, the most frequent site of residual conduction was the central anterior segment of the RPVs (24.2% of all gaps), followed by the central posterior segment (19.7%) and the central anterior segment of the LPVs (13.6%).

Serious adverse events occurred in three patients (1.9%) with no significant differences between the groups (1.3 vs. 2.6%, *P* = 0.99). In the by-LAWT group, a pseudoaneurysm at the femoral punction site requiring treatment with thrombin injection occurred in one patient. In the CLOSE group, one patient experienced a pseudoaneurysm at the femoral punction site requiring treatment with thrombin injection, while one patient experienced early post-procedural severe cardiac effusion, requiring pericardial drainage; the hospitalization of this patient was extended by 1 day, but without further complications. No neurological complications were observed, and no patient died. Three patients of the CLOSE group and one patient in the by-LAWT group experienced pericarditis within 15 days of the procedure, which did not require hospitalization, even if these events did not fall within the safety endpoint.

### Long-term outcomes

**Figure 4:**
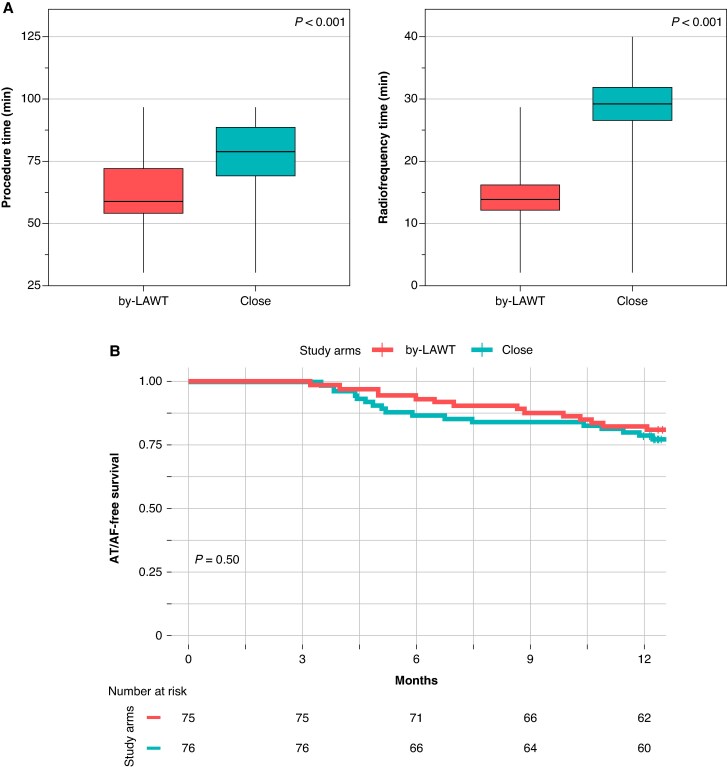
Procedural outcomes (*A*) and atrial arrhythmia-free survival Kaplan–Meier curves (*B*) according to the study arm.

At the time of ablation, most patients (82%) were on amiodarone therapy. Flecainide was used in two patients in the by-LAWT group and in one patient in the CLOSE group. All patients discontinued AADs after the 3 month blanking period, except those who experienced an arrhythmic recurrence during the blanking period. During the 3 month blanking period, arrhythmic recurrence occurred in 26 patients (16.7%), with no significant difference between groups (*P* = 0.83); all recurrences were managed with electrical cardioversion. Among these, 16 patients (10.3%) continued AADs beyond 6 months, with no differences between groups. Five patients (3.1%), three in the by-LAWT group and two in the CLOSE group, did not complete the 12-month follow-up and were excluded for the long-term analysis. At the end of the 12 month follow-up, 14 patients (18.7%) from the by-LAWT group and 17 (22.4%) from the CLOSE group experienced AT/AF recurrence (*P* = 0.50). Among the 31 patients who experienced arrhythmic recurrence, 7 presented with paroxysmal AF, 23 with persistent AF, and 1 with left atrial atypical flutter, with no differences between groups (*P* = 0.88). Of these, 15 patients underwent a second ablation procedure. Pulmonary vein reconnection was found in 12 of them (5 in the by-LAWT group and 9 in the CLOSE group), while in the remaining 3 patients, all pulmonary veins were found to be durably isolated (1 in the by-LAWT group and 2 in the CLOSE group), with no significant differences between the two groups (*P* = 0.99). No significant difference was observed in AT/AF recurrence rates between women and men (*P* = 0.5) nor within the female subgroup when comparing by-LAWT vs. CLOSE arms (*P* = 0.84). Procedural outcomes and atrial arrhythmia-free survival Kaplan–Meier curves according to the study arm are shown on *Figure 4*.

### LAWT-guided radiofrequency delivery

In the study cohort, anterior wall segments were thicker in the LPVs as compared to those in the RPVs [2.19 mm (1.57–2.83) vs. 1.60 mm (1.23–1.96), *P* < 0.01], while posterior wall segments had similar WT in LPVs and RPVs [1.16 mm (1.04–1.32) vs. 1.11 mm (0.90–1.39), *P* = NS]. In the by-LAWT arm, LAWT-guided AI delivery was significantly higher for the anterior segments of LPVs as compared to that of the RPVs [422 (383–464) vs. 390 (374–411), *P* < 0.01], while AI delivery was similar for posterior wall segments of LPVs and RPVs [331 (315–371) vs. 335 (320–352), *P* = NS].

With respect to the CLOSE arm, the by-LAWT arm reported significantly lower AI delivery at the anterior [422 (383–464) vs. 555 (525–570), *P* < 0.01] and posterior [331 (315–371) vs. 380 (360–410), *P* < 0.01] segments of the LPVs and at the anterior [390 (374–411) vs. 553 mm (535–565), *P* < 0.01] and posterior [335 (320–352) vs. 395 (375–425), *P* < 0.01] segments of the RPVs.

In the by-LAWT group, the oesophageal fingerprint overlapped with at least one segment of the posterior wall ablation line in 74 out of 78 patients (94.9%), except in the 4 patients where the oesophagus coursed purely along the central posterior wall without intersecting the ablation line. In only one patient, it was possible to modify the course of the ablation line to avoid the area of oesophageal contact and maintain the standard LAWT-guided AI delivery. In all other 73 patients, lower AI values (300 in the closest ‘red’ zone) were intentionally applied along the portion of the ablation line adjacent to the oesophageal fingerprint.

**Figure 5:**
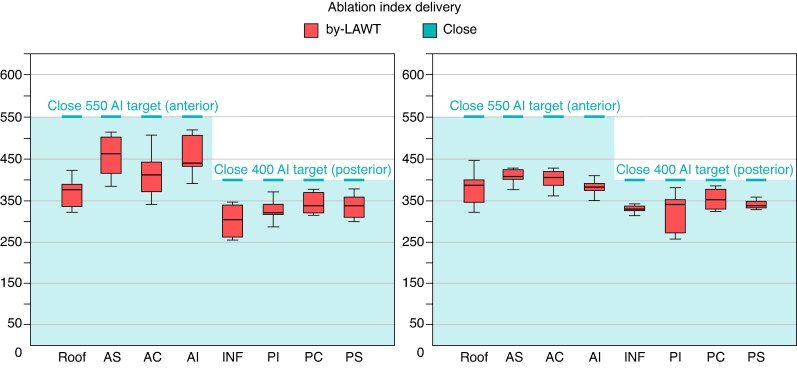
Box plots showing the personalized AI titration according to local left atrial wall thickness in the intervention arm, compared with the fixed AI targets of the CLOSE protocol. AC, anterior carina; AI, antero-inferior; AS, antero-superior; Inf, inferior; LPV, left pulmonary vein; PC, posterior carina; PI, postero-inferior; PS, postero-superior; RPV, right pulmonary vein.

Box plots illustrating the personalized AI titration based on local LAWT in the by-LAWT group, and its visual comparison with the fixed AI targets of the CLOSE protocol, are shown in *Figure 5*.

Ladybug plots of the mean LAWT for each PV segment are reported in the Supplementary material online, *Figure S1*.

## Discussion

### Main findings

This is the first prospective randomized study comparing the outcomes of an ablation protocol that adjust the radiofrequency delivery to the tissue thickness with the standard of care CLOSE protocol PVI for PeAF ablation. The main findings of the study are as follows: (i) a LAWT-guided PVI for PeAF ablation is associated with a high-rate recurrence-free survival at 12 month follow-up, demonstrating no differences compared to the results of PVI based on the CLOSE protocol; (ii) the LAWT-guided protocol proved to be more efficient with respect to the CLOSE protocol, demonstrating a significant reduction of procedure and radiofrequency times; (iii) the LAWT-guided approach for PeAF ablation demonstrated a first-pass PVI rate and major complication rate comparable to those of the CLOSE protocol, although the study was not powered to detect differences in these secondary endpoints.

### Procedure efficacy

A previous published proof-of-concept study^14^ showed that LAWT-guided PVI is feasible but also effective for patients with PeAF. This randomized controlled study confirmed these previous findings, demonstrating no statistically significant difference in the atrial arrhythmias recurrence rate at 12 month follow-up compared to PVI based on the CLOSE protocol. Maintenance of sinus rhythm at 12 months was 81.3% in the by-LAWT and 77.6% in the CLOSE group (*P* = 0.50). These results are in line with those previously reported by our group^14^ and with those reported in literature regarding PVI-only ablation in a cohort of patients with PeAF. In the recent PRAISE trial,^9^ clinical recurrence was documented in 20% of patients at the end of 12 month follow-up, while the STAR AF II trial reported a recurrence-free survival rate of 59% at 18 month follow-up for the PVI-only ablation arm.^7^ Although this study was not powered to assess sex-specific differences, we observed no significant difference in AT/AF recurrence rates between women and men (*P* = 0.5), nor within the female subgroup when comparing by-LAWT vs. CLOSE arms (*P* = 0.84).

### Procedure efficiency

Patients with PeAF differ in several key aspects from those with PAF, including greater LA remodelling and a higher prevalence of structural heart disease and comorbidities such as hypertension, diabetes, and left ventricular dysfunction.^5,29^ Catheter ablation for PeAF is more demanding procedure than for paroxysmal AF, requiring longer durations and greater amounts of radiofrequency energy delivery. This underscores the need for ablation strategies that improve procedural efficiency without compromising effectiveness. This need is especially relevant in the current era of pulsed field ablation and single-shot techniques, which have already demonstrated maintained efficacy while significantly reducing procedure times.^30^ In this context, the by-LAWT group achieved the intended outcomes with significantly less radiofrequency energy compared to the CLOSE protocol group. Furthermore, the by-LAWT group experienced a significant reduction in total procedure time, confirming the high efficiency of this personalized approach with respect to the standard strategy. These improvements can be attributed to LAWT-guided AI titration and the personalized ablation line, which aims to avoid thicker regions of the LA, whenever possible.

An extensive analysis of inter-subject and intra-anatomical variability in LAWT across the LA–PV junction has been previously published by our group.^12^ In that study, our group reported that wall thickness varies significantly not only between anterior and posterior regions but also across specific anatomic segments. For instance, the posterior superior segment of the LPVs displayed a mean LAWT of 0.7 ± 0.3 mm, while anterior segments such as the anterior central reached 2.2 ± 0.6 mm. Similar variability was observed in the RPVs. Importantly, this variability was evident not only across different segments but also between patients within the same segment of the antrum of the PVs. Understanding this variability in LAWT is crucial, as without LAWT maps, empirical prediction the ablation mode in a specific segment becomes challenging. Efficiency should be considered not only as a marker of procedural quality in terms of timesaving but also as a potential contributor to increased safety. Longer procedural times have generally been associated with a higher rate of complications, regardless of the ablation strategy employed.^31^ It is worth noting that, compared with previous literature, in this trial it was reported a reduction in procedure and fluoroscopy times also in the CLOSE study arm. For example, in the PRAISE trial,^9^ PeAF patients undergoing PVI-only radiofrequency ablation following the CLOSE protocol reported a procedure time of 158 ± 34 min, radiofrequency time of 36 ± 9 min, and fluoroscopy time of 12 min. Similarly, in the PVI-only subgroup of the STAR AF II trial,^7^ procedural outcomes included a procedure time of 167 ± 55 min and a fluoroscopy time of 29 ± 16 min. The shorter procedure and fluoroscopy times observed in this study, even in the CLOSE group, may be explained by the systematic use of HFLTV,^32^ a standardized stepwise near-zero fluoroscopy approach with TOE guidance,^23^ a single catheter approach,^24^ and a routinely performed pre-procedural imaging integration.^33,34^

### Safety outcomes

The present study lacks sufficient power to detect significant differences in safety outcomes between the two groups. Nevertheless, no radiofrequency-related adverse events, such as deaths, pericardial effusion, atrio-oesophageal fistulas, or symptomatic PV stenosis, were observed in the by-LAWT group. This finding supports the previous evidence of good safety profile of this approach in a cohort of PeAF patients.^14^ There are two main advantages supporting the use of cardiac imaging to personalize the AF ablation approach.^17^ First, imaging allows a precise localization of the areas with decreased wall thickness, potentially facilitating safer radiofrequency energy delivery. Excessive energy delivery in these regions may increase the risk of severe complications, such as atrial perforation or atrio-oesophageal fistula. Consequently, experts recommend using lower energy levels when ablating the thinnest areas, particularly along the posterior aspect of the LA.^16^ In this study, the total radiofrequency energy delivered was lower in the by-LAWT group, for both the anterior and the posterior aspect of the ablation line along the PV antrum. Second, MDCT image post-processing enables the generation of oesophageal isodistance maps, which can be integrated into the navigation system. This capability allows for a further personalization of the ablation lines, ensuring they are placed away from areas of the LA posterior wall where the atrio-oesophageal distance is shortest. Additionally, in cases where ablation in these regions is unavoidable, the information of a distance map allows for further reduction in the AI target at the nearest zones.^26^

Regarding radiation exposure, a dose-optimized ECG-gated protocol has been routinely employed in our institution since the implementation of the by-LAWT ablation programme. The administered radiation dose with this method is 160 mGy·cm, corresponding to an effective dose of 3 mSv and a total absorbed dose of approximately 27 mGy.^35^

### Clinical implications

This study was designed to test a purposely less demanding and more efficient ablation strategy, based on personalized AI delivery guided by local LAWT. While improved procedural efficiency was expected by design, what this trial demonstrates for the first time is that such an individualized approach can achieve clinical outcomes equivalent to those of the standard CLOSE protocol, in terms of both acute efficacy and 12 month arrhythmia-free survival. The rationale for not pursuing a superiority design in terms of recurrence-free survival stems from the findings of our prior pilot study,^14^ which showed encouraging 1 year arrhythmia-free survival rates comparable to those achieved with the CLOSE protocol, but without sufficient evidence to support a superiority hypothesis in terms of efficacy. Nonetheless, the results of the present trial are positive, as they demonstrate that a personalized LAWT-guided strategy successfully meets the goal of achieving similar efficacy to a validated standard approach while significantly improving procedural efficiency—an increasingly important objective in contemporary ablation practice. The main advantage of a LAWT-guided ablation strategy lies in the potential to reduce the total amount of radiofrequency energy delivered, leading to shorter procedure time, while maintaining overall efficacy, with comparable acute and long-term outcomes to the standard of care and without an increase in adverse events, although the study was not powered to detect differences in safety outcomes. The rationale behind these good results lies in tailoring the AI to the tissue thickness, ending up with a totally personalized ablation strategy. Previous studies have identified minimum AI values of 540 and 380 as predictors of freedom from acute reconnection in the anterior/roof and the posterior/inferior segments of the PV antrum, respectively.^27^ However, growing evidence highlights the complex anatomy of the LA, including intra- and inter-patient variability in the distribution of LAWT at both the anterior and posterior segments of the PV–LA junction.^36^ Indeed, in the present study, LAWT ranged from 0.56 to 2.33 mm in the posterior wall and from 0.85 to 4.54 mm in the anterior wall. Therefore, relying on fixed AI targets and a dichotomized anterior/posterior ablation approach may be an oversimplification, as it does not fully account for this variability. Eventually, this may lead to non-transmural lesions in the thickest areas of the LA and, conversely, excessive radiofrequency energy application in the thinnest regions. The proposed approach of tailoring AI based on the local LAWT allows to personalize the radiofrequency delivery: increasing radiofrequency energy in the thickest areas of the lesion line enhancing lesion transmurality while reducing energy delivery in the thinnest areas. Although the present study focused on the PVI alone, the clinical application of LAWT-based ablation may extend beyond PVI, particularly in patients with PeAF who require adjunctive substrate modification. This method could support more rational placement and dosing of linear lesions, reducing the risk of incomplete lines and reconnection. Future studies are needed to validate this hypothesis and to determine whether LAWT-guided linear ablation could improve outcomes beyond those achieved in earlier trials such as STAR AF.^7^

In the participating centres, where pre-procedural MDCT and the imaging post-processing software are routinely used for all AF ablation procedures, the only additional cost is software availability. As this randomized trial was specifically designed to compare procedural and clinical outcomes, no formal cost-effectiveness analysis was conducted in this study.

## Limitations

The main limitation of the study is that AI targets for each LAWT range were based on empirical data already used in several published studies^12–15,26^; further research will be needed to define the optimal AI parameters according to LAWT, potentially evaluating whether a more aggressive AI setting in selected thick-walled regions of the LA–PV junction—where reconnection has been reported to occur more frequently^14^—may enhance durability and clinical results. Second, due to the procedural nature of the intervention, operators could not be blinded to treatment allocation, which may have introduced bias; however, endpoint adjudication was performed in a blinded fashion to mitigate this risk. Third, all procedures were performed under general anaesthesia, mechanical ventilation with HFLTV, and a single catheter approach; thus the generalizability of the findings might be limited. Fourth, we adopted the classical 3 month blanking period, but we cannot exclude that a shorter blanking period might be preferable.^37^ Fifth, the study population included few patients with longstanding PeAF and was characterized by overall low CHA₂DS₂-VASc scores and only moderately dilated left atria, which may limit generalizability to higher-risk populations. However, given the emerging trend towards earlier intervention in contemporary ablation trials, our cohort may reflect a representative subset of modern clinical practice.^38,39^ Finally, in recent years the use of continuous or high-frequency monitoring via wearable devices (e.g. photoplethysmography- or ECG-enabled smartwatches) has emerged as a promising tool to improve arrhythmia detection after catheter ablation.^28,40^ In the absence of this daily monitoring, it is possible that brief asymptomatic arrhythmic episodes were missed,^41,42^ potentially leading to an overestimation of success rates; however, both study arms would be equally affected. Future studies describing the outcomes of by-LAWT may benefit from integrating wearable-based monitoring to improve the diagnostic sensitivity and granularity of arrhythmia outcome data.

## Conclusions

The PeAF-by-LAWT trial is the first prospective randomized study to demonstrate that a personalized LAWT-guided PVI for PeAF ablation is non-inferior to the standard CLOSE protocol in terms of arrhythmia-free survival while significantly improving procedural efficiency. While safety outcomes were comparable between groups, the study was underpowered to detect statistically significant differences in complication rates.

## Supplementary Material

euaf163_Supplementary_Data

## Data Availability

The data that support the findings of this study are available from the corresponding author, upon reasonable request.
