# Supplementary figures and images for: Personalized pulmonary vein isolation guided by left atrial wall thickness for persistent atrial fibrillation ablation: the PeAF-by-LAWT randomized trial
Source: Europace. 2025 Aug 6;27(12):euaf163. doi: 10.1093/europace/euaf163 (PMC12676948; doi:10.1093/europace/euaf163)

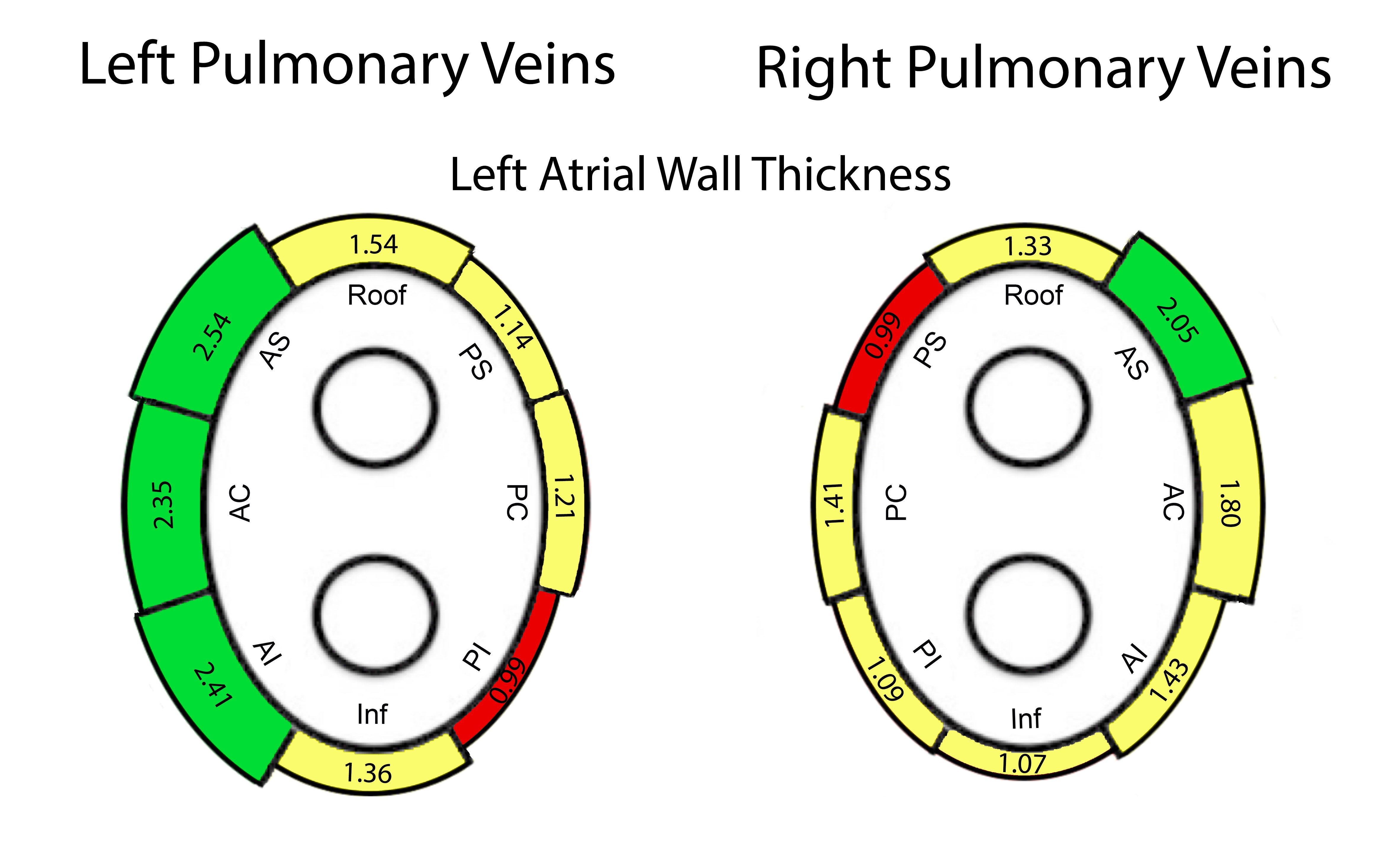

Supplement: euaf163_Supplementary_Data [file euaf163_supplementary_data.jpeg]
